# 2025 Egyptian guidelines for the management of dyslipidemia

**DOI:** 10.1186/s43044-026-00716-9

**Published:** 2026-01-20

**Authors:** Ashraf Reda, Hesham S. Taha, Hala Mahfouz Badran, Hazem Khamis, Sherif Kamal, Ahmed Shawky Elserafy, Muhammad Hasan Dawoud, Ahmed Adel Elamragy, Ahmed Bendary, Mirna Mamdouh Shaker

**Affiliations:** 1 Menoufia University, Shibīn al-Kawm, Egypt; 2 Cairo University, Giza, Egypt; 3 Wady El Nile Hospital, Cairo, Egypt; 4 Egyptian health authority, Cairo, Egypt; 5 Ain Shams University, Cairo, Egypt; 6 Benha University, Benha, Egypt

**Keywords:** Dyslipidemia, Atherogenesis, LDL-cholesterol, Lipid-lowering therapies, Patient risk categories

## Abstract

**Background:**

The burden of premature atherosclerotic cardiovascular disease (ASCVD) in Egypt remains disproportionately high, and current international dyslipidemia guidelines have proven insufficient in achieving target lipid levels in the local population. This underscores the necessity for a context-specific national guideline. The 2025 Egyptian Guidelines for the Management of Dyslipidemia were developed through a structured consensus process led by an expert panel of cardiologists, endocrinologists, and representatives from national medical societies.

**Main text:**

The methodology included two rounds of blind voting followed by a consensus meeting to ensure rigorous evaluation. The guidelines emphasize the importance of early detection of dyslipidemia through systematic screening programs and prioritize lifestyle interventions as the cornerstone of management. A novel “extreme-risk” category was introduced to identify patients requiring intensified lipid-lowering strategies, including early initiation of combination pharmacotherapy. Additionally, the recommendations highlight the critical role of ongoing monitoring and follow-up to sustain long-term lipid control and reduce cardiovascular risk.

**Conclusion:**

The current guidelines provide a simplified, yet evidence-based framework tailored to the Egyptian population, aiming to optimize dyslipidemia management, reduce ASCVD-related complications, and improve overall cardiovascular outcomes.

**Supplementary Information:**

The online version contains supplementary material available at 10.1186/s43044-026-00716-9.

## Introduction

Dyslipidemia represents a major global health concern, with recent data indicating a prevalence of nearly 40% among Egyptians, particularly higher in urban populations due to lifestyle and dietary factors [1]. Sedentary behavior, obesity, genetic predisposition, and nutritional transitions over the past decades have contributed substantially to this burden.

Pathophysiologically, the “response-to-retention” hypothesis describes atherosclerosis initiation as the subendothelial retention of cholesterol-rich apoB-containing lipoproteins under conditions of endothelial dysfunction. Prolonged exposure to elevated plasma lipoproteins, particularly low-density lipoprotein cholesterol (LDL-C), accelerates atherosclerotic progression, with cumulative burden correlating directly with clinical risk [2]. Other apoB-containing particles, including lipoprotein(a) [Lp(a)] and cholesterol-rich remnants of triglyceride-rich lipoproteins, further potentiate vascular injury [3].

Given the escalating burden of atherosclerotic cardiovascular disease (ASCVD) in Egypt, the development of national dyslipidemia guidelines is essential. Such guidance ensures context-specific risk stratification, screening, and therapeutic strategies, optimizes resource utilization, and reduces variability in clinical practice [4]. Moreover, standardized recommendations foster early detection, public awareness, and research opportunities, ultimately improving cardiovascular outcomes and public health.

## Purpose

These recommendations aim to establish a nationally agreed framework for the management of dyslipidemia in Egypt, addressing the high prevalence of premature atherosclerotic cardiovascular disease (ASCVD) and the limited success in achieving lipid targets under existing international guidelines. The document provides evidence-based, context-specific guidance for early detection strategies, lifestyle interventions, and pharmacological lipid-lowering therapy, with particular emphasis on individuals at elevated or extreme risk. In addition, the guidelines underscore the importance of structured monitoring and long-term follow-up to ensure sustained lipid control and improved cardiovascular outcomes within the Egyptian population.

## Scope

- Focuses on the management of dyslipidemia within the Egyptian population.
- Incorporates local epidemiological characteristics and healthcare system challenges.
- Provides a standardized, evidence-based framework to guide clinical practice.
- Aims to reduce variability in treatment approaches and ensure consistency in care.
- Serves as an educational and reference tool to support healthcare providers in professional development and decision-making.

## Target audience

- Healthcare professionals managing cardiovascular risk:
- Cardiologists
- Endocrinologists
- Primary care physicians
- Healthcare policymakers and public health officials develop national prevention and treatment strategies.
- Researchers working on dyslipidemia and cardiovascular disease in Egypt.

## Executive summary

The 2025 Egyptian Dyslipidemia Guidelines provide evidence-based recommendations tailored to the local population and healthcare context, addressing the high prevalence of premature ASCVD and the suboptimal achievement of lipid-lowering targets under international standards. Developed through a structured consensus process, the guidelines encompass early detection strategies, cardiovascular risk assessment, lifestyle modification, and pharmacological management.

Screening recommendations emphasize risk evaluation at different age intervals, with particular attention to individuals with a positive family history or other major cardiovascular risk factors. Lifestyle advice includes adopting a Mediterranean-style diet rich in fruits, vegetables, legumes, nuts, fish, and whole grains, while limiting red and processed meats, refined carbohydrates, salt, and saturated fats. Regular physical activity, weight control, and smoking cessation are highlighted as cornerstones of non-pharmacological management.

Pharmacological therapy is detailed across the spectrum of available agents, including statins, ezetimibe, PCSK9 inhibitors, fibrates, omega-3 fatty acids, bempedoic acid, lomitapide, mipomersen, and ANGPTL3 inhibitors. Treatment algorithms specify LDL-C reduction goals, use of combination therapy for patients not meeting targets, and aggressive lipid lowering in those categorized as extreme risk. Cardiovascular risk assessment is recommended using SCORE2 and SCORE2-OP models, with explicit criteria for identifying extreme-risk patients and guiding management intensity.

Monitoring protocols include lipid profile assessment 4–12 weeks after therapy initiation or adjustment, followed by 6–12-month intervals depending on risk status. Liver enzymes and creatine kinase should be evaluated when indicated to detect adverse effects.

Overall, these national guidelines aim to unify clinical practice, improve long-term lipid control, reduce ASCVD-related complications, and enhance cardiovascular outcomes in Egypt.

## Methods

### Review of evidence

A comprehensive literature search was conducted by the core group of experts to gather evidence on dyslipidemia management, cardiovascular risk assessment, and lipid-lowering therapies relevant to the Egyptian context. The search included peer-reviewed original articles, randomized clinical trials, review articles, systematic reviews, and meta-analyses published in English until July 2024. The utilized electronic databases included PubMed, Medline, Cochrane Library, Embase, and Web of Science. The key search terms included “dyslipidemia,” “cardiovascular disease,” “lipid-lowering therapy,” “LDL-C,” “Egypt,” “familial hypercholesterolemia,” and “atherosclerosis.” Articles were screened based on titles and abstracts, and full texts were reviewed to ensure relevance and quality of evidence. References for selected articles were also screened to identify further studies.

### Consensus development

The recommendations were developed using a modified Delphi consensus process involving twenty national experts in cardiology (Supplementary Table 1), internal medicine, nephrology, and clinical pharmacy. An initial draft, based on literature review and international guidelines, was refined through two rounds of voting (3-point Likert scale) and an in-person consensus meeting (Supplementary Tables 2–4. Agreement was defined as ≥75%. Final statements were reviewed by all panelists for clarity, accuracy, and clinical applicability.

## Recommendations

### Cardiovascular risk assessment

#### Total cardiovascular risk and stratification

Total cardiovascular (CV) risk reflects the probability of an atherosclerotic event based on combined risk factors. The panel supports ESC stratification [5] but recommends distinguishing extreme risk (e.g., acute coronary syndrome [ACS]) from very high risk (e.g., chronic coronary syndrome [CCS]), as ACS patients have the highest recurrence risk, particularly in the first year [6], and require intensified management (Table 1) [5].

**Table 1:** Key recommendations for cardiovascular risk assessment in Egypt

Population/category	Recommendation
Children (2–8 years)	Lipid screening in the presence of a family history of familial dyslipidemia, premature ASCVD, or major risk factors (e.g., diabetes, hypertension, obesity)
Children (9–11 years)	Universal lipid screening at least once
Adolescents (17–21 years)	Universal lipid screening at least once
Adults (≥20 years)	Initial risk factor evaluation at the first clinical encounter; initiate lifestyle modification and/or treatment as indicated
Adults (40–69 years)	Use SCORE2 to estimate 10-year cardiovascular risk in apparently healthy individuals without established ASCVD, diabetes, CKD, or genetic/rare lipid disorders
Adults (≥70 years)	Use SCORE2-OP for risk estimation in apparently healthy individuals without ASCVD, diabetes, CKD, or genetic/rare lipid disorders
Extreme-risk criteria*	Subset of very high-risk patients who should be managed as *extreme risk* Multiple recurrent CV events (esp. within 2 years) One major CV event + ≥2 high-risk conditions (smoking, diabetes, hypertension, CKD) Polyvascular disease Multivessel coronary artery disease Recent ACS (<12 months)
Moderate risk	Re-evaluate using risk modifiers before final risk classification

*ASCVD* atherosclerotic cardiovascular disease, *CAD* coronary artery disease, *ACS* acute coronary syndrome, *CKD* chronic kidney disease

In individuals without ASCVD, diabetes, or chronic kidney disease (CKD), risk should be estimated using SCORE2 (ages 40–69) or SCORE2-OP (≥70), which provide 10-year risk based on WHO-calibrated country clusters [7]. These models are accessible via the ESC CVD Risk tool and regional charts but are not suitable for those with established CVD, familial hypercholesterolemia, rare lipid disorders, CKD, or pregnancy [7]. A specific SCORE2-DM algorithm has been introduced for diabetes [8]. In moderate-risk patients, additional modifiers (Table 2) may support reclassification and initiation of lipid-lowering therapy.

**Table 2:** Risk modifiers for cardiovascular disease

Domain	Risk modifiers
Genetic & family	Premature ASCVD (men < 55, women < 65); Familial dyslipidemia; Lp(a) > 50 mg/dL
Metabolic/systemic	Metabolic syndrome (MetS); chronic kidney disease (CKD); NAFLD; hs-CRP > 2.0 mg/L
Inflammatory/immune	Chronic immune-mediated inflammatory conditions
Cardiovascular	Left ventricular hypertrophy; Atrial fibrillation; Carotid/femoral atherosclerosis; ABI < 0.9
Respiratory	Obstructive sleep apnea
Neurological	Migraine with aura
Psychosocial	Psychosocial factors
Gender-specific	Premature menopause (<40 years); Pregnancy-related hypertension; Preeclampsia/eclampsia; Erectile dysfunction
Diagnostic markers	Coronary artery calcium (CAC) score > 0 AU

*ASCVD* atherosclerotic cardiovascular disease, *Lp(a)* lipoprotein (a), *NAFLA* non-alcoholic fatty liver disease, *hs-CRP* high-sensitive C-reactive protein, *ABI* Ankle-Brachial index

Egypt is classified as a very high-risk country, with cardiovascular disease (CVD) accounting for 46.2% of non-communicable disease-related deaths in 2017 [9]. The Egyptian CardioRisk project (2020) further demonstrated that 51% of Egyptians presented with premature acute coronary syndrome (ACS), while dyslipidemia affected 48.2% of individuals [10–12]. These data highlight the need for systematic cardiovascular risk assessment. Current guidance recommends evaluation of risk factors (Table 2), including lipid screening, during the initial visit in adults ≥ 20 years, with re-evaluation as indicated. For children, universal lipid screening is advised at ages 9–11 and again at 17–21 years, while targeted screening from age 2 is recommended for those with a family history of dyslipidemia or premature ASCVD [13]. Prompt recognition and timely management of these high-risk patients can help lessen the incidence of subsequent cardiovascular events [14].

### Lipids and lipoproteins measurement

Measurement of lipids and lipoproteins plays a central role in assessing the risk of ASCVD and guiding therapeutic decisions (Table 3), taking into consideration the following rules:

- Use fasting or non-fasting lipid profile (TC, TG, HDL-C, LDL-C, non-HDL-C) for ASCVD risk assessment.
- Repeat fasting profile if TG ≥ 400 mg/dL, or in familial dyslipidemia/high TG.
- Measure Lp(a) once in adulthood; identify very high inherited levels (>180 mg/dL).
- Consider Lp(a) in premature ASCVD, recurrent vascular events, or as a risk modifier in moderate/high-risk patients.

**Table 3:** Lipid and lipoprotein measurements in ASCVD risk assessment

Parameter/method	Key points	Recommendation/notes	References
LDL-C (Friedewald formula)	Traditional calculation: TC—(TG/5)—HDL-C	Inaccurate in MetS, DM, HTG	[15]
LDL-C (Direct enzymatic)	Overcomes limitations of Friedewald	Current method of choice	[15]
Non-HDL-C	TC—HDL-C; reflects all ApoB-containing atherogenic lipoproteins	Useful alternative when ApoB not available	[16]
ApoB	Superior analytical performance vs. LDL-C/non-HDL-C	Limited by cost and availability	[16]
Lipoprotein(a) [Lp(a)]	High levels ↑ lifetime ASCVD risk; reclassifies moderate risk; relevant in premature or recurrent ASCVD despite optimal LDL-C/non-HDL-C	Measure at least once in lifetime	[17]
Fasting vs. non-fasting samples	Both acceptable, similar prognostic value	Fasting required in familial dyslipidemia or TG > 400 mg/dL; otherwise, non-fasting practical	[18]

*LDL-C* low-density lipoprotein-cholesterol, *TC* total cholesterol, *TG* triglycerides, *HDL-C* high-density lipoprotein-cholesterol, *MetS* metabolic syndrome, *DM* diabetes mellitus, *HTG* hypertriglyceridemia, *ApoB* apolipoprotein B, *ASCVD* atherosclerotic cardiovascular diseases

### Therapeutic goals and regimens

**Fig. 1:**
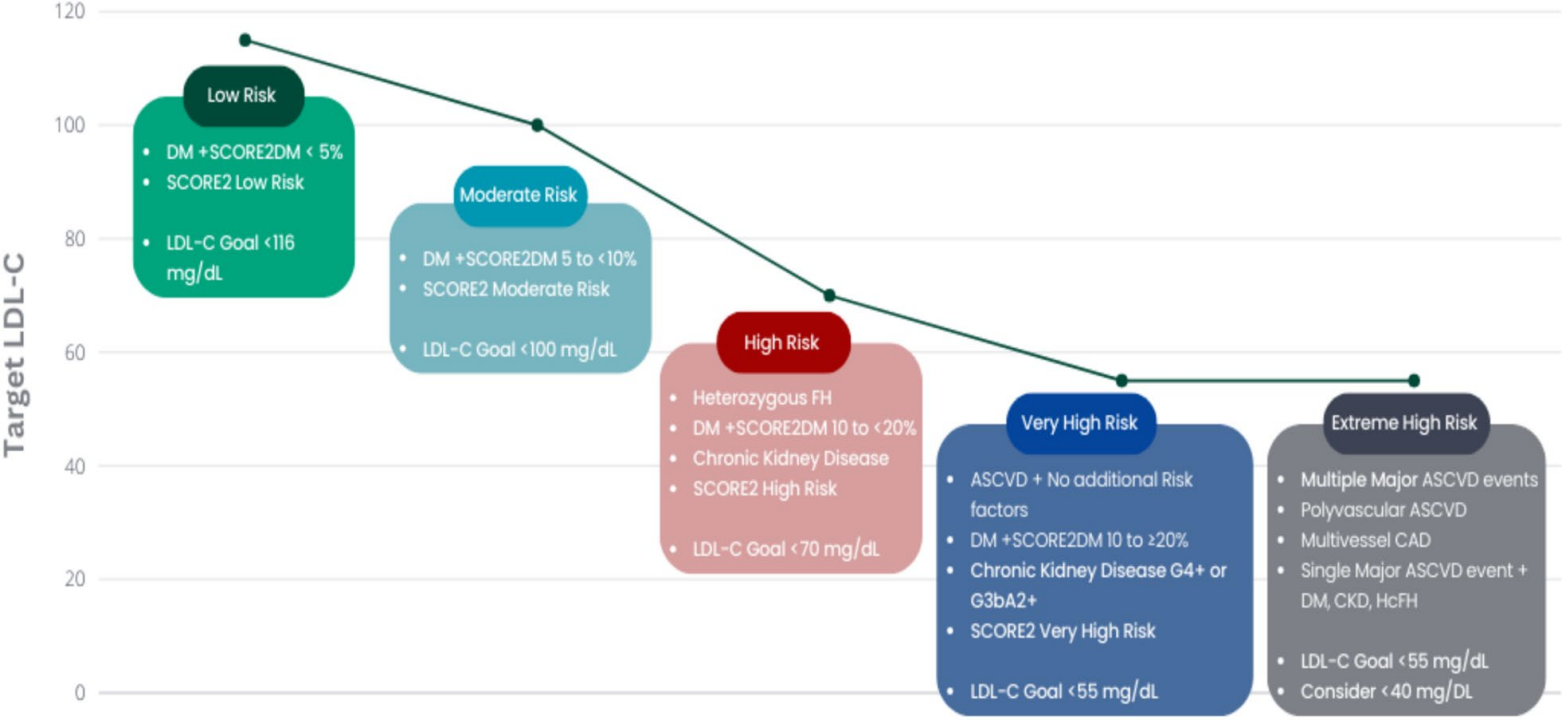
Risk categories & LDL-C goals. DM, diabetes mellitus; SCORE2DM, systematic coronary risk evaluation 2 DM; LDL-C, low-density lipoprotein cholesterol; FH, familial hypercholesterolemia; ASCVD, atherosclerotic cardiovascular disease; SCORE2, systematic coronary risk evaluation 2; CAD, coronary artery disease; CKD, chronic kidney disease; HeFH, heterozygous FH

Patients at higher cardiovascular (CV) risk gain the most from intensive treatment [19]. Lifestyle changes are essential for both primary and secondary prevention [4]. Statins are first-line therapy for hypercholesterolemia in ASCVD, with non-statin agents considered when targets are unmet or statins are not tolerated [20]. Ezetimibe, PCSK9 inhibitors, and bempedoic acid effectively lower LDL-C and CV events [21], while inclisiran is being evaluated in outcome studies [22]. Early combination therapy is increasingly recommended to maximize LDL-C reduction, tolerability, and adherence (Table 4; Fig. 1) [23].

**Table 4:** Summary of LDL-C and Non-HDL-C goals

Risk category	LDL-C goal	Additional notes
Extreme high risk	<55 mg/dL and ≥50% reduction from baseline. Consider lowering LDL-C < 40 mg/dL	Lifestyle modification + intensive therapy; non-HDL-C < 85 mg/dL
Very high risk	<55 mg/dL and ≥50% reduction from baseline	Lifestyle modification + intensive therapy; non-HDL-C < 85 mg/dL
High risk	<70 mg/dL and ≥50% reduction from baseline	Non-HDL-C < 100 mg/dL
Moderate risk	<100 mg/dL	Non-HDL-C < 130 mg/dL
Low risk	<116 mg/dL (may be considered)	

*LDL-C* low-density lipoprotein-cholesterol,* non-HDL-C* non-high-density lipoprotein-cholesterol

#### General recommendations

- Lifestyle modification for all (primary & secondary prevention).
- Low/moderate risk with LDL-C < 190 mg/dL → 3-month lifestyle trial, then recheck.

### Monitoring & safety in lipid-lowering therapy

**Fig. 2:**
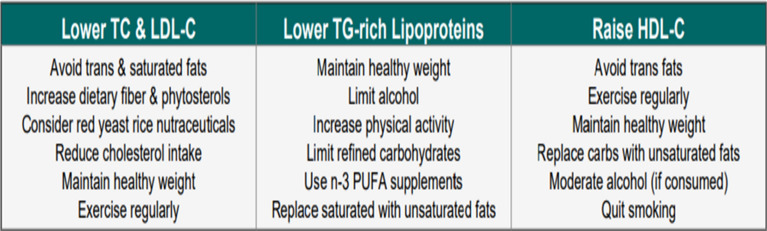
Lifestyle strategies for optimizing lipid profile. TC, total cholesterol; LDL-C, low-density lipoprotein-cholesterol; TG, triglyceride; HDL-C, high-density lipoprotein-cholesterol; *n* − 3 PUFA, omega-3 polyunsaturated fatty acid

Before initiating statin therapy, patients should be evaluated for factors predisposing to adverse effects, including advanced age, comorbidities, prior statin‐associated muscle symptoms (SAMS), history of myalgia, potential drug interactions, and risk of new-onset diabetes [24]. Baseline measurement of alanine aminotransferase (ALT) and creatine kinase (CK) is advised, with repeat ALT testing after 4–6 weeks; further monitoring is unnecessary unless abnormalities occur [5, 13]. Lipid profile assessment is recommended 4–12 weeks after starting or modifying therapy, followed by periodic evaluation—every 6 months in very high-risk individuals and annually in others. Guideline recommendations vary: the ESC advises reassessment every 6–12 months (earlier at 4–6 weeks post-ACS), whereas the ACC/AHA suggests intervals of 3–12 months [5, 13]. In patients at extreme cardiovascular risk, closer early follow-up is warranted (Table 5; Fig. 2). Continuous attention to adherence and lifestyle modification remains crucial for optimal ASCVD prevention.

**Table 5:** Monitoring of lipids and enzymes in patients on lipid-lowering therapy

Domain	When to assess	Action/thresholds
Lipid profile	4–12 weeks after initiation/change (4–6 weeks in ACS); then q6mo (extreme risk) or annually (others)	Full lipid panel + non-HDL-C as secondary target
ALT (liver)	Baseline + 4–6 weeks after start/dose change	<3× ULN → continue & recheck; ≥3× ULN → stop/reduce, recheck; cautious re-initiation if normalized
CK (muscle)	Baseline + if symptoms	<4× ULN (no symptoms) → continue; <4× ULN (with symptoms) → monitor/stop if persistent; 4–10× ULN (asymptomatic) → Re-check CK measurement, assess for secondary causes, consider discontinuation, recheck, & consider benefit/risk ratio; 4–10× ULN (symptomatic) → stop & monitor; >10× ULN → stop, check renal function, monitor CK q2w
Statin intolerance	During therapy adjustments	Try lower dose, alternate-day dosing, or statin + ezetimibe; partial intolerance → moderate-intensity statin + ezetimibe; complete intolerance → ezetimibe + bempedoic acid ± PCSK9 inhibitor
Other labs	HbA1c/glucose annually in diabetes-risk patients	Particularly elderly, MetS, obesity, insulin resistance, high-dose statins
Special notes	Throughout treatment	Consider transient CK rise (exercise, injections); monitor symptoms closely

*Non-HDL-C* non-high-density lipoprotein-cholesterol,* ALT* alanine transaminase, *ULN* upper limit of normal,* CK* creatine phosphokinase,* PCSK9* proprotein convertase subtilisin/kexin type 9, *MetS* metabolic syndrome

### Management of dyslipidemia

#### Lifestyle goals for cardiovascular disease prevention

Avoiding tobacco is essential. A heart-healthy diet emphasizing fruits, vegetables, whole grains, nuts, legumes, fish, and vegetable oils while limiting red/processed meat, refined carbs, salt, and animal fats lowers CV risk. The PREDIMED trial showed that a Mediterranean diet with olive oil or nuts reduced major CV events by 30% [25].

Saturated fats raise LDL-C (0.8–1.6 mg/dL per 1% energy), and trans fats worsen LDL-C and HDL-C; replacing them with unsaturated oils lowers LDL-C. Saturated fat should be <10% of energy (<7% if hypercholesterolemia), with cholesterol ≤ 300 mg/day. MUFAs and PUFAs, including fish ≥2 times/week and plant-based *n* − 3 sources, reduce CV mortality and stroke [26].

Carbohydrates should be 45–55% of energy. Alcohol, if used, should be ≤ 100 g/week; and avoid completely in hypertriglyceridemia and in hepatic disease. At least 150 min/week of activity, BMI 20–25 kg/m^2^, and waist < 94 cm (men) or <80 cm (women) is advised [ 27].

#### Lipid-lowering drugs (Table 6)

The general theraputic approach to LDL-C reduction is demonstratedin Table 6.

**Table 6:** Therapeutic approach to LDL-C reduction according to risk level

Patient group/risk	Initial therapy	If LDL-C target not achieved	further options/notes
General/indicated patients	Statin at highest tolerated dose	Add ezetimibe	Consider PCSK9 inhibitor for very-high-risk or FH patients
Statin-intolerant patients	Bempedoic acid + ezetimibe	PCSK9 inhibitor if needed	Individualization based on LDL-C response
Elderly, CKD, partial statin intolerance	Moderate-intensity statin + ezetimibe	Escalate therapy as needed	Stepwise escalation depending on LDL-C
Extremely high ASCVD risk	High-intensity statin + ezetimibe	If additional LDL-C lowering <20% → bempedoic acid; >20% → PCSK9 inhibitor	Target LDL-C <55 mg/dL; consider <40 mg/dL individually

*LDL-C* low-density lipoprotein-cholesterol, *PCSK9* proprotein convertase subtilisin/kexin type 9, *FH* familial hypercholesterolemia, *CKD* chronic kidney disease, *ASCVD* atherosclerotic cardiovascular disease

*Statins*. The lipid-lowering effect of statins is dose-dependent and varies among different agents (Table 7). High-intensity regimens generally reduce LDL-C by >50%, while moderate-intensity therapy achieves a 30–50% reduction. Statins also lower triglycerides by 10–20% and raise HDL-C modestly (1–10%), depending on drug and dose [28]. Their impact on lipoprotein(a) [Lp(a)] is negligible or occasionally increases levels [29]. Beyond lipid effects, statins exert anti-inflammatory and antioxidant actions [30].

**Table 7:** LDL-C reductions (%) with high-, moderate-, and low- intensity statin therapy

	High intensity	Moderate intensity	Low intensity
LDL-C lowering	≥50%	30–49%	<30%
Statins	Atorvastatin 40–80 mg Rosuvastatin 20–40 mg	Atorvastatin 10–20 mg Rosuvastatin 5–10 mg Simvastatin 20–40 mg Pravastatin 40–80 mg Lovastatin 40–80 mg Fluvastatin XL 80 mg Fluvastatin 40 mg BID Pitavastatin 1–4 mg	Simvastatin 10 mg Pravastatin 10–20 mg Lovastatin 20 mg Fluvastatin 20–40 mg

*LDL-C* low-density lipoprotein-cholesterol

The Cholesterol Treatment Trialists (CTT) meta-analysis, involving >170,000 subjects across 26 RCTs, demonstrated that each 1 mmol/L LDL-C reduction with statin therapy corresponded to a 22% drop in major vascular events, 23% fewer major coronary events, 20% lower CAD mortality, 17% reduction in stroke, and 10% decrease in overall mortality over 5 years [31].

**Fig. 3:**
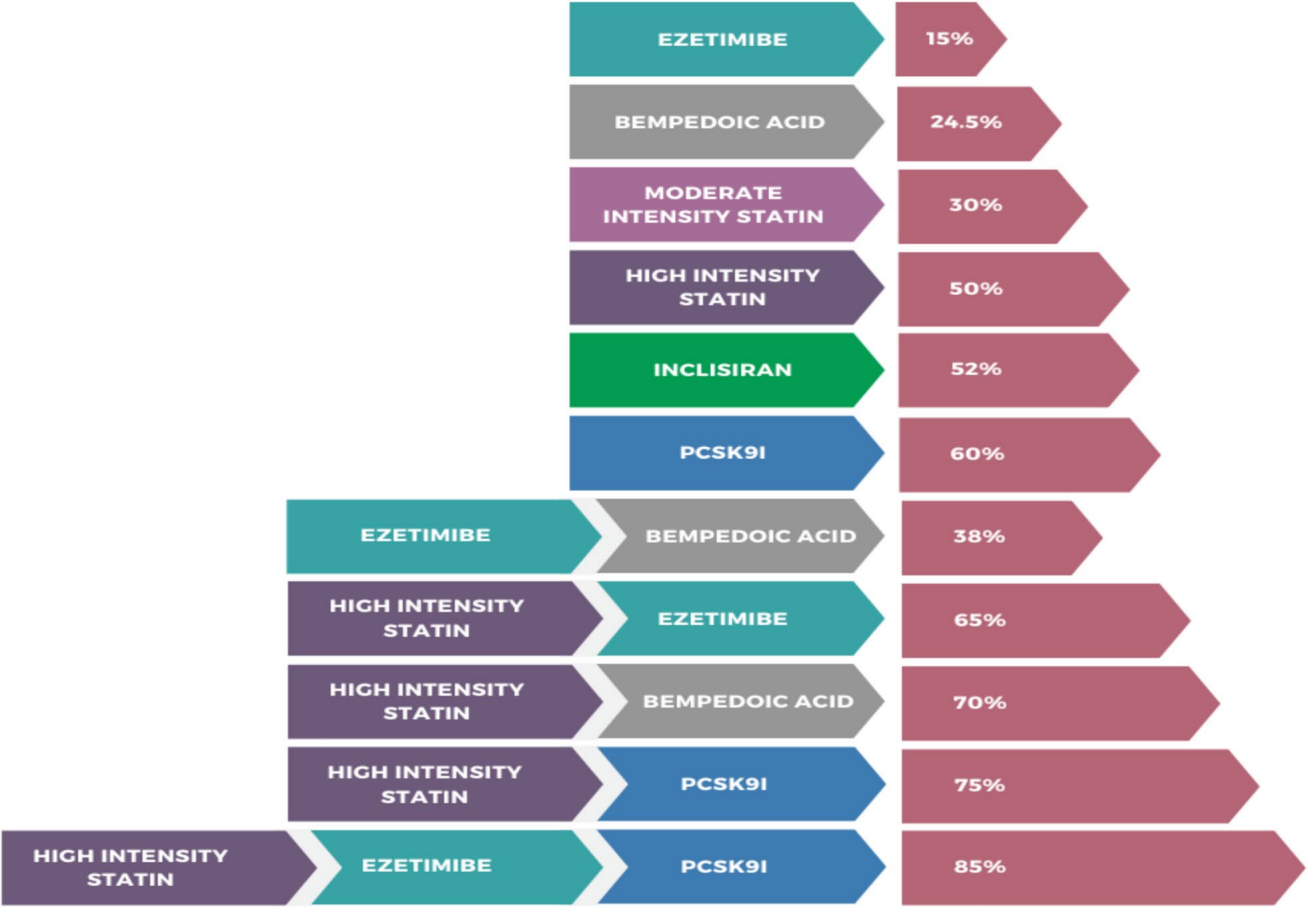
Lipid lowering therapies, combination therapy and the corresponding reduction in LDL-C. PCSK9I, proprotein convertase subtilisin/kexin type 9 inhibitor

Statins are usually well tolerated. Adverse effects include muscle-related symptoms (reported in 5–20% of patients, with true intolerance being rare), mild ALT elevation in 0.5–2%—mainly at higher doses—while severe hepatic injury is uncommon [32]. They slightly increase type 2 diabetes risk, particularly in older adults or those with metabolic predisposition, but the cardiovascular benefit clearly outweighs this risk. Data on hemorrhagic stroke remain inconsistent and require further clarification. Patients not reaching LDL-C goals or unable to use statins may require additional non-statin therapies (Fig. 3) [33].

*Cholesterol absorption inhibitors*. Ezetimibe acts at the intestinal brush border by inhibiting the Niemann–Pick C1-like protein 1 (NPC1L1), reducing uptake of both dietary and biliary cholesterol. This decreases cholesterol transport to the liver, leading to upregulation of LDL receptors and enhanced clearance of circulating LDL particles. When used alone at 10 mg/day, it typically lowers LDL-C by about 15–22%, though the response varies among individuals. A review of RCTs (>2700 patients) reported an 18.5% LDL-C reduction versus placebo, along with modest improvements in HDL-C (+3%), TGs (−8%), and TC (−13) [34]. In the IMPROVE-IT trial, nearly 18,000 post-ACS patients were randomized to simvastatin alone or with ezetimibe; after 7 years, combination therapy reduced CV events compared with statin alone (32.7% vs. 34.7%, *p* = 0.016) [35].

*Bile acid sequestrants*. Cholestyramine, colestipol, and colesevelam are bile acid-binding resins that inhibit enterohepatic reabsorption of bile acids. Depletion of bile acids stimulates hepatic conversion of cholesterol into bile acids, thereby upregulating LDL receptor expression and lowering circulating LDL-C. Daily doses (24 g cholestyramine, 20 g colestipol, or 4.5 g colesevelam) reduce LDL-C by 18–25%, with minimal effect on HDL-C but potential TG elevation in susceptible individuals. Their use is limited by gastrointestinal adverse effects (flatulence, constipation, dyspepsia, nausea) and possible reduction in fat-soluble vitamin absorption [36].

*Proprotein convertase subtilisin/kexin type 9 (PCSK9) targeted therapy*. PCSK9 promotes lysosomal degradation of LDL receptors (LDLR), reducing their expression and increasing circulating LDL-C. PCSK9-targeted therapies include monoclonal antibodies (alirocumab, evolocumab) and the siRNA-based agent inclisiran, which inhibits PCSK9 gene expression to enhance LDLR recycling [37]. Alirocumab and evolocumab reduce LDL-C by ~60% in clinical trials, while inclisiran achieves ~50% LDL-C reduction and a 26% decrease in Lp(a) [37, 38].

Two major outcome trials established the cardiovascular efficacy of PCSK9 inhibitors. In FOURIER, involving 27,564 ASCVD patients on background statin therapy, evolocumab reduced median LDL-C from 92 to 30 mg/dL and lowered major adverse cardiovascular events by 15% over 2.2 years (HR 0.85, 95% CI 0.79–0.92) [39]. In ODYSSEY Outcomes, 18,924 patients with recent ACS treated with alirocumab achieved a decline in LDL-C from 92 to 48 mg/dL, with a 15% reduction in the composite outcome over 2.8 years (HR 0.85, 95% CI 0.78–0.93) [40]. Outcome studies with inclisiran, such as ORION-4, are still underway [41]. The main adverse events reported are injection-site reactions and influenza-like symptoms [42].

*Fibrates. Mechanism*: Activate peroxisome proliferator-activated receptor-α (PPAR-α), regulating transcription of genes involved in lipid metabolism and promoting TG-rich lipoprotein catabolism. *Effect*: Reduce TG by ~50%, modestly lower LDL-C (<20%, though LDL-C may rise when baseline TG is high), and increase HDL-C by <20% [43]. *Key trials*: *Helsinki Heart Study (HHS)*: Gemfibrozil reduced CVD outcomes [44]. *FIELD and ACCORD*: Fenofibrate did not reduce overall CVD outcomes [43, 45]. *LEADER*: Bezafibrate showed no significant reduction in composite CVD outcomes [46]. *Fenofibrate* slowed progression of diabetic retinopathy by 30–40% over 4–5 years in patients with type 2 diabetes and pre-existing retinopathy [43]. *Safety*: Generally well tolerated; GI side effects (<5%) and skin rashes (~2%) reported. Risks include myopathy (especially with statins), elevated liver enzymes, and cholelithiasis. Fibrates also increase serum creatinine and homocysteine, though creatinine levels usually normalize after discontinuation [47].

*Omega-3 fatty acids. Mechanism*: Eicosapentaenoic acid (EPA) and docosahexaenoic acid (DHA) reduce TG synthesis, likely via PPAR activation and decreased ApoB secretion. *Effect*: Lower triglycerides by up to 45%, with minimal impact on LDL-C or HDL-C. *Key trials*: Meta-analysis of 79 trials (112,059 participants): No significant effect on total mortality (RR 0.98, 95% CI 0.90–1.03) or CV events (RR 0.99, 95% CI 0.94–1.04); slight reduction in CHD events (RR 0.93, 95% CI 0.88–0.97) [48]. *ASCEND*: 15,480 diabetics without ASCVD—no reduction in vascular events with *n* − 3 supplementation (RR 1.00, 95% CI 0.91–1.09) [49]. *REDUCE-IT*: 8000 statin-treated patients with elevated TGs—EPA lowered major adverse CV events by 25% vs. placebo [50]. *Safety*: Generally well tolerated; adverse effects include GI upset. May increase bleeding risk when combined with antiplatelets [51].

*Bempedoic acid. Mechanism*: Inhibits ATP citrate lyase, reducing hepatic cholesterol synthesis upstream of HMG-CoA reductase and upregulating LDL receptor activity. *Effect*: Reduces LDL-C by up to 18% when added to maximally tolerated statin therapy; lowers hs-CRP but has minimal effect on TGs or Lp(a). *Key trial*: CLEAR Outcomes (~14,000 patients, primary and secondary prevention): Bempedoic acid reduced LDL-C by 18% and lowered risk of MACE by 21% (including MI, stroke, and CV death) [52]. *Safety*: Common adverse effects include hyperuricemia and upper respiratory tract infections. Rare cases of tendon rupture were reported; discontinue immediately if rupture occurs and avoid in patients with tendon disorders [52].

#### Other lipid-lowering therapies

*Lomitapide*. Lomitapide is an effective agent for lowering lipid levels, mainly prescribed for individuals with homozygous familial hypercholesterolemia (HoFH). Its mechanism involves blocking microsomal triglyceride transfer protein (MTP), thereby limiting the formation and release of apolipoprotein B-containing lipoproteins from both the intestine and liver. This action produces a marked reduction in circulating LDL cholesterol. However, treatment can be complicated by gastrointestinal intolerance, as well as the risk of hepatic steatosis and elevated liver enzymes, necessitating regular monitoring. Because of these concerns, lomitapide is typically reserved for patients with very high cholesterol who do not respond adequately to other available therapies [53].

*Mipomersen*. Mipomersen is an antisense oligonucleotide therapy designed to lower lipid levels in patients with HoFH. It functions by inhibiting the synthesis of apolipoprotein B-100, a key protein in the production of LDL-C. Mipomersen significantly decreases LDL-C levels in patients who are inadequately controlled with conventional lipid-lowering treatments. However, its use is often limited by side effects such as injection site reactions, flu-like symptoms, and potential liver enzyme elevations, necessitating regular monitoring during therapy [54].

*Angiopoietin-like protein 3 (ANGPTL3)*. Angiopoietin-like protein 3 (ANGPTL3) is a promising target in lipid-lowering therapy, particularly for patients with familial hypercholesterolemia and other severe lipid disorders. Inhibitors of ANGPTL3 function by reducing the activity of lipoprotein lipase and endothelial lipase, leading to decreased levels of LDL-C, TGs, and HDL-C. Emerging therapies, such as monoclonal antibodies and RNA-based drugs targeting ANGPTL3, have demonstrated significant lipid-lowering effects and are well-tolerated in clinical trials [55].

### Special considerations in dyslipidemia management

#### Elderly

Statin therapy is recommended for secondary prevention in elderly patients aged > 65 years with ASCVD, and for primary prevention in those <75 years similarly to younger adults. However, evidence supporting initiation of statins for primary prevention in individuals >75 years is limited [56]. CVD remains a major cause of disability in people aged ≥ 65 years, reducing their health span by approximately 6 years [57]. Clinical trials have shown that statins significantly reduce major vascular events in patients with established vascular disease regardless of age, but the benefits are less evident in individuals > 75 years without ASCVD [13]. In addition, safety concerns must be considered, as comorbidities, altered pharmacokinetics and pharmacodynamics, and polypharmacy in the elderly may increase the risk of muscle-related adverse effects and drug interactions [58].

#### Children and adolescents

Lifestyle modifications, including a healthy diet and regular aerobic exercise, are first-line recommendations for children and adolescents with lipid disorders. Shared decision-making with families is crucial, ensuring appropriate counseling, education, and safety considerations before initiating pharmacotherapy (Table 8) [59]. Drug therapy is generally reserved for patients aged ≥ 8–10 years with severe, persistent hypercholesterolemia (LDL-C ≥ 190 mg/dL, or ≥160 mg/dL) with familial hypercholesterolemia or a family history of premature CAD after secondary causes have been excluded. The therapeutic goal is a ≥50% LDL-C reduction or achieving levels < 130 mg/dL, ideally < 110 mg/dL [60]. FDA-approved statins for pediatric use include rosuvastatin and pravastatin from age 8 years, and atorvastatin, simvastatin, lovastatin, and fluvastatin from age 10 years, while cholestyramine may be used from age 6 years [61]. PCSK9 inhibitors have expanded therapeutic options: evolocumab is approved for patients ≥ 10 years with heterozygous FH, showing a 38% LDL-C reduction at 12 weeks in the HAUSER-RCT trial without major safety concerns [62], and alirocumab has recently been approved from age 8 years as an adjunct to statins with reassuring safety data [63]. For children with triglycerides ≥ 500 mg/dL, who are at risk for pancreatitis, fibrates and/or omega-3 fatty acids may be considered despite no FDA-approved options, preferably under specialist guidance [64].

**Table 8:** Pediatric lipid classification (mg/dL) [65]

	Acceptable (mg/dL)	Borderline (mg/dL)	High (mg/dL)
Total cholesterol	<170	170–199	≥200
LDL-C	<110	110–129	≥130
Non-HDL	<120	120–144	≥145
Triglycerides (0–9) years	<75	75–99	≥100
Triglycerides (10–19) years	<90	90–129	≥130
HDL-C	>45	40–45	N/A

*LDL-C low-density lipoprotein-cholesterol, non-HDL-C non-high-density lipoprotein-cholesterol, HDL-C high-density lipoprotein-cholesterol*

#### Women

In women, lipid-lowering drugs are contraindicated during pregnancy, breastfeeding, and when planning pregnancy. In severe cases, bile acid sequestrants or LDL apheresis may be considered under specialist care [66]. Estrogen replacement therapy is not recommended for CV prevention, while low-dose third-generation oral contraceptives are acceptable only in women with normal LDL-C; alternative contraception is advised if LDL-C > 160 mg/dL or with multiple CV risk factors [67]. Meta-analyses, including 174,000 participants, and the IMPROVE-IT trial confirmed similar benefits of statins and ezetimibe in both sexes [31, 35]. Although the FDA lifted the universal pregnancy contraindication in 2021, current guidelines advise discontinuing statins at least 2 months before conception and avoiding them in pregnancy and lactation until further safety data emerge [68]. High-risk women, such as those with homozygous FH or ASCVD, should be referred to a lipid specialist [4, 13].

#### Diabetes mellitus

In patients with DM, the most common phenotype of dyslipidemia includes a combination of high plasma TG concentrations, low HDL-C levels, and increased levels of small, dense LDL-C particles. LDL-C is the primary target for lipid-lowering therapy in these patients. To manage their risk appropriately, patients with DM should be assessed using the SCORE-DM tool [69].

#### Acute coronary syndromes (ACS)

Lipid-lowering therapy is integral in ACS management. High-intensity statins, started early regardless of baseline LDL-C, reduce recurrent ischemic events and MACE, especially within the first 16 weeks [70]. Lower-intensity statins may be used in elderly patients or those with renal/hepatic impairment or drug interactions [5]. Lipid profile should be rechecked at 4–6 weeks, with goals of ≥50% LDL-C reduction and ≤55 mg/dL [71]. If not achieved, add ezetimibe; in very high-risk patients, initiate statin/ezetimibe combination upfront. If targets remain unmet after 4–6 weeks, add PCSK9 inhibitors [13]. Pre-treatment with high-intensity statins before PCI is recommended to reduce peri-procedural MI, MACE, and contrast-induced kidney injury [72].

#### Stroke

High-intensity statins are recommended for patients with ischemic stroke or TIA, with benefits comparable to aspirin or antihypertensive therapy [73]. Each 1 mmol/L (39 mg/dL) LDL-C reduction lowers stroke risk by 21% and recurrent stroke by 17%, while also reducing major CV events [74]. Early statin use after TIA or stroke is crucial, with particular benefit in patients with carotid stenosis [75]. Concerns about hemorrhagic stroke are minimal, with no proven causal link, and benefits outweigh potential risks [76].

#### Peripheral arterial disease (PAD)

High-intensity statins are recommended for PAD, an independent risk factor for MI and CV mortality. If LDL-C ≤ 55 mg/dL is not achieved with statins, ezetimibe and PCSK9 inhibitors should be added [4]. Station reduces CV events by ~20% in LEAD [77], while evolocumab lowers CV events and major limb events by 42% in PAD [39]. Fenofibrate also reduces amputation risk, likely via non-lipid mechanisms [43].

### Management of hypertriglyceridemia

Hypertriglyceridemia (HTG) affects 10–29% of adults and may be primary (genetic) or secondary to alcohol, obesity, metabolic syndrome, DM, hypothyroidism, renal disease, pregnancy, SLE, or certain drugs. Abnormal TG is >175 mg/dL (non-fasting) or >150 mg/dL (fasting); severe HTG is >500 mg/dL and very severe > 1000 mg/dL. Statins, ezetimibe, and PCSK9 inhibitors modestly lower TG (5–15%), while fibrates and omega-3 fatty acids achieve greater reductions (25–45%). LDL-C-lowering therapies reduce ASCVD risk regardless of TG level. Management starts with correcting secondary causes and lifestyle, especially limiting alcohol. High-risk patients should receive statins first-line. In statin-treated patients with TG 135–499 mg/dL, icosapent ethyl (2 g twice daily) is recommended; for TG > 200 mg/dL despite LDL-C control, fenofibrate or bezafibrate may be added. For primary prevention in low–moderate risk patients with TG > 200 mg/dL, omega-3 fatty acids may be considered [4, 78, 79].

### Familial dyslipidemias

Genetic factors significantly influence plasma lipid levels, with familial hypercholesterolemia (FH) being the most common inherited dyslipidemia strongly linked to CVD. FH is usually suspected with markedly elevated LDL-C and classified under Fredrickson types IIa, IIb, and III, with type IIa being the most common. Mutations on chromosome 19 impair LDL receptor function, reducing LDL uptake and raising cholesterol levels; these mutations may be heterozygous or homozygous, determining disease severity and age of onset [80]. Table 9 summarizes the genetic disorders of lipoprotein metabolism.

**Table 9:** Genetic disorders of lipoprotein metabolism

Disorder	Prevalence	Gene(s)	Effect on lipoproteins
HeFH	1/200–250	LDLR, APO B, PCSK9	↑LDL-C
HoFH	1/160,000–320,000	LDLR, APO B, PCSK9	↑↑LDL-C
FCH	1/100–200	USF1 + modifying genes	↑LDL-C, ↑VLDL-C, ↑ApoB
Familial dysbetalipoproteinemia	1/5000	APO E	↑↑IDL & chylomicron remnants (βVLDL)
Familial lipoprotein lipase deficiency	2/1,000,000	LPL, APO C2, ApoAV, GPIHBP1, LMF1	↑↑chylomicrons & VLDL-C
Tangier disease (analphalipoproteinemia)	1/1,000,000	ABCA1	↓↓HDL-C
Familial LCAT deficiency	1/1,000,000	LCAT	↓HDL-C

*Apo* apolipoprotein, *FCH* familial combined hyperlipidemia, *HDL-C* high-density lipoprotein cholesterol, *HeFH* heterozygous familial hypercholesterolemia, *HoFH* homozygous familial hypercholesterolemia, *IDL* intermediate-density lipoprotein, *LCAT* lecithin cholesterol acyltransferase, *LDL-C* low-density lipoprotein cholesterol, *VLDL* very low-density lipoprotein cholesterol

#### Heterozygous familial hypercholesterolemia

Heterozygous familial hypercholesterolemia (HeFH) carries a tenfold higher CHD risk, with untreated patients often developing CAD before age 55 in men and 60 in women. Early detection and treatment markedly lower this risk. HeFH affects ~1 in 200–250 individuals (≈14–34 million worldwide), yet most remain undiagnosed and undertreated [81].

#### Homozygous familial hypercholesterolemia

Homozygous familial hypercholesterolemia (HoFH) is a rare, life-threatening disorder (1:160,000–320,000) marked by severe LDL-C elevation, xanthomas, and premature CVD. CAD and aortic stenosis often occur before age 20, with death before 30, necessitating early detection and referral to lipid specialists [5].

Diagnostic criteria include untreated LDL-C > 10 mmol/L (>400 mg/dL), xanthomas before age 10, and/or elevated LDL-C in both parents. Genetic confirmation involves bi-allelic pathogenic variants in LDLR, APOB, PCSK9, or LDLRAP1. Most cases arise from LDLR (85–90%), followed by APOB (5–10%) and PCSK9 (1–3%) variants. The rare autosomal recessive hypercholesterolemia (ARH) (<1%) results from biallelic LDLRAP1 loss-of-function [82].

Family history of premature CAD and physical findings (tendon xanthomas, corneal arcus < 45 years, tuberous xanthomas, xanthelasma) support diagnosis. Diagnostic tools include the Dutch Lipid Clinic Network, Simon Broome, and WHO criteria (Table 10) [83].

**Table 10:** Dutch Lipid Clinic Network diagnostic criteria for familial hypercholesterolemia

Category	Criteria	Points
Family history	1st-degree relative with premature CAD/vascular disease or LDL-C > 95th percentile	1
	1st-degree relative with xanthomata/arcus cornealis, or child (<18 years) with LDL-C > 95th percentile	2
Clinical history	Premature CAD (men < 55 years; women < 60 years)	2
	Premature cerebral/peripheral vascular disease	1
Physical exam^a^	Tendinous xanthomata	6
	Arcus cornealis before age 45	4
LDL-C (untreated)	≥8.5 mmol/L (≥325 mg/dL)	8
	6.5–8.4 mmol/L (251–325 mg/dL)	5
	5.0–6.4 mmol/L (191–250 mg/dL)	3
	4.0–4.9 mmol/L (155–190 mg/dL)	1
DNA analysis	Pathogenic mutation in LDLR, apoB, or PCSK9	8

Choose only one score per group, the highest applicable; diagnosis is based on the total number of points obtained A ‘definite’ FH diagnosis requires >8 points, probable’ FH diagnosis requires 6–8 points ‘possible’ FH diagnosis requires 3–5 points

*CAD* coronary artery disease, *FH* familial hypercholesterolemia, *LDL-C* low-density lipoprotein cholesterol, *PCSK9* proprotein convertase subtilisin/kexin type 9

^a^Exclusive of each other (i.e., maximum 6 points if both are present)

Before diagnosing HoFH, clinicians must exclude secondary causes of high LDL-C (nephrotic syndrome, biliary cirrhosis, hypothyroidism, anorexia, medications) and consider differentials such as sitosterolemia, cerebrotendinous xanthomatosis, polygenic hypercholesterolemia, familial combined hyperlipidemia, hyperapobetalipoproteinemia, and dysbetalipoproteinemia [84].

FH complications include premature CAD, MI, HF, stroke, aortic stenosis, PAD, and CV death. Even with statins, FH patients face higher risks: post-ACS mortality is doubled, and CAD/death risk reaches 52% in male and 32% in female relatives. HoFH has a poor prognosis, often fatal before age 30 [85].

Management requires a holistic approach: lifestyle modification (diet, exercise, smoking cessation, weight control) and family education. Statins at maximal doses are first-line, followed by ezetimibe (10–30% LDL-C reduction) and PCSK9 inhibitors (50–60%) [39]. In resistant cases, mipomersen, lomitapide, lipoprotein apheresis, or surgical approaches (ileal bypass, liver transplantation) may be required [86]. LDL goals depend on risk level, with monitoring every 2–3 months to adjust therapy [13].

Managing FH requires a multidisciplinary team (primary care, cardiology, endocrinology, lipid specialists, dietitians, pharmacists, nurses) and education programs to improve awareness. Patients with persistently high LDL-C despite maximal statin therapy should be referred to specialized lipid clinics. Lifestyle counseling (diet, weight control, smoking cessation) and education on CVD risk and risk factor management are essential, and first-degree relatives should undergo screening [85].

#### Familial combined hyperlipidemia

Familial Combined Hyperlipidemia (FCH) is the most common mixed dyslipidemia (1:100–200), marked by elevated LDL-C, TGs, or both, and a major cause of premature CAD. Diagnosis is often missed; clues include ApoB > 120 mg/dL, TGs > 133 mg/dL, and family history of premature CVD. Mixed dyslipidemia underscores the need for intensified treatment, as high TGs add to the risk from elevated LDL [87].

#### Familial dysbetalipoproteinemia

Familial Dysbetalipoproteinemia (Type III Hyperlipoproteinemia) is a rare AR disorder (variable penetrance) characterized by elevated TC and TG (7–10 mmol/L) and accelerated atherosclerosis, especially in femoral and tibial arteries. Clinical signs include tubero-eruptive and palmar xanthomas. It usually appears later in life and rarely in premenopausal women. The condition is strongly linked to ApoE2 homozygosity, impairing hepatic clearance of chylomicron remnants and IDL. Diagnosis requires ApoE2 confirmation, and management should occur in specialized lipid clinics [88].

#### Genetic causes of hypertriglyceridemia

Hypertriglyceridemia (HTG) has a complex genetic basis. Polygenic variants impairing VLDL synthesis and clearance usually cause moderate TG elevations (2–10 mmol/L), while rare monogenic forms disrupt chylomicron clearance, leading to severe HTG, chylomicronemia, pancreatitis, and lipid deposition. Mutations in six AR genes—LPL, APOC2, APOA5, LMF1, GPIHBP1, and GPD1—have been identified [89].

#### Other genetic disorders of lipoprotein metabolism

Rare genetic HDL-C disorders include Tangier disease (analphalipoproteinemia) and LCAT deficiency, both marked by very low HDL-C and distinctive clinical syndromes requiring referral to specialized lipid clinics. In contrast, CETP deficiency results in markedly elevated HDL-C (80–90 mg/dL in heterozygotes; ≥200 mg/dL in homozygotes) but is not associated with ASCVD and may even be protective [90].

## National dyslipidemia recommendations

National guidelines emphasize lifestyle modification and risk-based lipid-lowering therapy, prioritizing the identification of patients at *very high* or *extreme* CV risk for targeted, personalized interventions. Initial combination therapy is encouraged in these groups, and bempedoic acid is suggested for patients not achieving LDL-C goals with statin/ezetimibe or in cases of statin intolerance, particularly in resource-limited settings. These user-friendly recommendations aim to optimize treatment and improve outcomes.

## Implementation considerations

The Egyptian dyslipidemia guideline panel emphasized the need for multiple strategies to ensure effective adoption and improved cardiovascular outcomes. Training programs, workshops, and continuous medical education should equip healthcare providers—including cardiologists, endocrinologists, primary care physicians, and allied health staff—with up-to-date evidence-based practices. On a policy level, the guidelines can support health authorities in integrating dyslipidemia management into national health plans, ensuring access to medications, screening, and comprehensive care. They also provide a framework for resource allocation. Public awareness initiatives, including educational campaigns, are crucial to promoting early detection and lifestyle modification (healthy diet, physical activity, smoking cessation, and regular lipid screening).

## Research needs

The Egyptian dyslipidemia guidelines highlight several research priorities, including epidemiological studies to clarify prevalence, risk factors, and regional patterns of dyslipidemia. Genetic studies are needed to better define predispositions to dyslipidemia and CVD in the Egyptian population, supporting personalized therapies. Further research should also evaluate the efficacy and safety of combination treatments, the cost-effectiveness of screening and management strategies, and factors influencing patient adherence to medications and lifestyle modifications.

The expert panel emphasized the importance of monitoring the implementation of the Egyptian dyslipidemia guidelines through key performance indicators, including screening rates, treatment adherence, and cardiovascular outcomes. Suggested measures include systematic data collection, regular audits, assessment of provider compliance, and patient feedback. Long-term outcome evaluations, policy reviews, and stakeholder engagement are essential for ongoing improvement, while research initiatives and regular reporting should be promoted to share best practices and success stories.

## Supplementary Information

Additional file 1.

## Data Availability

No datasets were generated or analysed during the current study.

## Abbreviations

ABI: Ankle-Brachial index
ACCORD: Action to control cardiovascular risk in diabetes
ACS: Acute coronary syndrome
ALT: Alanine transaminase
ANGPTL3: Angiopoietin-like protein 3
ApoB: Apolipoprotein B
ASCVD: Atherosclerotic cardiovascular disease
BMI: Body mass index
CAC: Coronary artery calcium
CAD: Coronary artery disease
CCS: Chronic coronary syndrome
CI: Confidence interval
CK: Creatine kinase
CKD: Chronic kidney disease
CV: Cardiovascular
CVD: Cardiovascular disease
CTT: Cholesterol treatment trialists
DHA: Docosahexaenoic acid
DM: Diabetes mellitus
EPA: Eicosapentaenoic acid
FIELD: Fenofibrate intervention and event lowering in diabetes
FH: Familial hypercholesterolemia
GI: Gastrointestinal
HDL-C: High-density lipoprotein cholesterol
HeFH: Heterozygous familial hypercholesterolemia
HHS: Helsinki Heart Study
HoFH: Homozygous familial hypercholesterolemia
HR: Hazard ratio
hs-CRP: High-sensitivity C-reactive protein
LDL-C: Low-density lipoprotein cholesterol
LDLR: Low-density lipoprotein receptor
LEADER: Liraglutide effect and action in diabetes: evaluation of cardiovascular outcome results
LLT: Lipid-lowering therapy
Lp(a): Lipoprotein(a)
MACE: Major adverse cardiovascular events
MTP: Microsomal triglyceride transfer protein
*n* − 3: Omega-3 fatty acids
NPC1L1: Niemann–Pick C1-like protein 1
ORION 4: A specific outcome trial for inclisiran
PCSK9: Proprotein convertase subtilisin/kexin type 9
PPAR-a: Peroxisome proliferator-activated receptor alpha
PREDIMED: Prevención con Dieta Mediterránea
PUFA: Polyunsaturated fatty acid
REDUCE-IT: Reduction of cardiovascular events with icosapent ethyl–intervention trial
SASE: Statin-associated side effects
SAMS: Statin-associated muscle symptoms
SCORE2: Systematic coronary risk evaluation 2
SCORE2-OP: SCORE2-older persons
SFA: Saturated fatty acid
SLE: Systemic lupus erythematosus
T2DM: Type 2 diabetes mellitus
TC: Total cholesterol
TGs: Triglycerides
USF1: Upstream transcription factor 1
VLDL: Very low-density lipoprotein
