# Supplementary material for: 2025 Egyptian guidelines for the management of dyslipidemia
Source: Egypt Heart J. 2026 Jan 20;78:6. doi: 10.1186/s43044-026-00716-9 (PMC12819915; doi:10.1186/s43044-026-00716-9)
Supplement: Supplementary file 1 — Additional file 1. [file 43044_2026_716_MOESM1_ESM.docx]

| Name | Title |
| --- | --- |
| Dr. Ahmed Adel ElAmragy | Associate Professor of Cardiology at Cairo University |
| Dr. Ahmed Mahmoud Bendary | Associate Professor of Cardiology at Benha University |
| Dr. Ahmed Mesbah | Consultant Cardiologist at the National Heart Institute |
| Dr. Ahmed Mohamed Onsy | Professor of Cardiology at Ain Shams University |
| Dr. Ahmed Shawky Elserafy | Professor of Cardiology at Ain Shams University |
| Dr. Ashraf Reda (Chairman) | Professor of Cardiology at Menofia University |
| Dr. Ebtisam Abd ElShaheed | Consultant of Internal Medicine at the Egyptian Healthcare Authority - Ismailia |
| Dr. Gamila Mohamed | Professor of Cardiology at Suez Canal University |
| Dr. Hazem Khamis | Professor of Cardiology at MTI University |
| Dr. Heba Kamal | Consultant of Clinical Pharmacy at the Egyptian Healthcare Authority |
| Dr. Hesham Salah Eldin Taha (Chairman) | Professor of Cardiology at Cairo University |
| Dr. Marwa Oraby | Consultant Nephrologist in Private Sector |
| Dr. Merihan Mohamed | Consultant Cardiologist at Elnasr Specialized Hospital |
| Dr. Mirna Mamdouh Shaker | Lecturer of Cardiology at Cairo University |
| Dr. Mohamed Hassan Dawoud | Associate Professor of Cardiology at Cairo University |
| Dr. Mona Karem | Associate Professor of Nephrology at Suez Canal University and Consultant at EHA |
| Dr. Seham Azmy | Consultant of Internal Medicine at Ismailia Medical Complex |
| Dr. Sherif Kamal | Chairman Advisor at the Egyptian Health Authority, Cairo, Egypt |
| Dr. Wessam ElShafeei | Professor of Cardiology at Menoufia University |

**Supplementary Table 1: Expert panel of voting in management dyslipidemia guidelines**

**Supplementary Table 2: Cardiovascular Risk Assessment**

| Recommendations |
| --- |
| Consider lipid screening at least once between ages 9 and 11. |
| Consider lipid screening later again between ages 17 and 21. |
| Consider lipid screening at age 2-8 years in children with a positive family history of familial dyslipidemia and/or premature ASCVD or other cardiovascular risk factors such as diabetes, hypertension, obesity, or other conditions that accelerate atherosclerotic risk. |
| Consider risk factor evaluation to assign risk status during the first patient visit for adults aged 20 years and older. Depending on the results, initiate efforts to control risk factors with re-evaluation or initiate lifestyle changes. |
| In Egypt, use the very high-risk SCORE2 tools to calculate the estimated CVD risk within the next 10 years for apparently healthy people aged 40-69 years (and SCORE2-OP for older individuals) without established ASCVD, DM, CKD, and genetic/rarer lipid disorders. |
| Patients at very high risk with at least one of the following criteria should be considered at extreme CVD risk: 1) multiple major CV events, especially if recurrent within two years, 2) one major CV event and 2 or more high-risk conditions such as current smoking, DM, HTN, and CKD, 3) polyvascular disease, 4) multivessel CAD, and 5) recent ACS within the past 12 months. |
| For patients in the moderate risk category, it is advisable to check for risk modifiers. |

**Supplementary Table 3: Monitoring of Lipids and Enzymes in Patients on Lipid-Lowering Therapy**

| Recommendations |
| --- |
| Assess the response to pharmacological lipid-lowering therapy 4-12 weeks after initiation or adjustment of therapy to evaluate treatment effectiveness and any necessary adjustments. In acute coronary syndrome, assessment of the response should be done earlier, after 4-6 weeks. |
| After a stable LDL-C goal is achieved, regular lipid monitoring is recommended every six months for patients at extreme CV risk and annually for patients in the other risk groups. |
| During follow-up, a full lipid profile should be performed. Additionally, non-HDL-C should also be assessed and used as a secondary treatment target. |
| Before starting statin therapy, patients should be evaluated for potential predisposing factors for statin-associated side effects. |
| Alanine transaminase (ALT) should be routinely measured in patients on statins before treatment, and in those with normal baseline levels, it should be repeated once 4-6 weeks after starting drug treatment or after a dose increase. Otherwise, routine ALT measurement is not recommended unless symptoms suggesting liver disease evolve. Control of ALT is still recommended during treatment with fibrates. |
| If ALT rise is less than 3 times the upper limit of normal, continue therapy and re-check liver enzymes in 4-6 weeks. |
| If ALT rise is 3 or more times the upper limit of normal, discontinue lipid-lowering therapy or decrease dose and re-check liver enzymes within 4-6 weeks. Cautious re-initiation of therapy may be considered after ALT has returned to normal with frequent rechecking of ALT levels. |
| Creatine kinase (CK) should be routinely measured in patients who will receive statins before treatment and after dose increases. If the baseline CK rise is more than four times the upper limit of normal, do not initiate drug therapy and re-check. Otherwise, routine CK measurement is not recommended unless the patient develops myalgia. |
| If CK becomes elevated in a person taking lipid-lowering drugs, first re-evaluate the indication for statin treatment. Further management would depend on whether the patient has muscle symptoms and the degree of CK elevation. |
| If CK rises >10 fold the upper limit of normal, discontinue treatment, check renal function, and monitor CK every 2 weeks. |
| If CK levels rise between 4 and 10 times the upper limit of normal and the patient is symptomatic, discontinue statin and monitor CK. |
| If CK levels rise between 4 and 10 times the upper limit of normal and the patient is asymptomatic, re-check CK measurement, assess for secondary causes, and consider discontinuing the statin, reducing the dose, or switching to a different statin, with follow-up of CPK. Continuing the statin while re-checking CK within 2 to 6 weeks may be considered. However, the continuation of therapy is a call of judgment depending on multiple factors, including the patient's overall level of risk. |
| If CK rises <4 fold the upper limit of normal, and the patient is not symptomatic, continue statin, explain to the patient to be alert about muscle symptoms, and re-check CK. |
| If CK rises <4 fold the upper limit of normal, and the patient has muscle symptoms, monitor symptoms and CK regularly. If symptoms persist, discontinue statin and re-evaluate symptoms after 2-6 weeks. |
| If CK normalizes after statin discontinuation, consider re-challenge with the same or another statin, low-dose statin, alternate day or once/twice weekly dosing regimen, or combination therapy with a lower statin dose or an alternative drug. |
| If a statin-based regimen is not tolerated at any dosage, even after rechallenge, bempedoic acid/ezetimibe combination should be considered, with the possible addition of PCSK9 inhibitor, if the goal is not achieved. |
| In patients who are elderly, at risk of myopathy, have CKD, or have partial statin intolerance, consider the initiation with a combination of moderate-intensity statin and ezetimibe. |
| In the case of complete statin intolerance, an initial combination of ezetimibe and bempedoic acid can be considered. Further addition of PCSK9 targeted therapy may be needed according to the LDL-C level achieved. Consider the possibility of transient CK elevation for other reasons, such as exertion or intramuscular injections. If CK remains elevated, consider myopathy. |
| HbA1c or blood glucose should be checked at least annually in patients at high risk of developing diabetes, such as the elderly, patients with metabolic syndrome, obesity, or other signs of insulin resistance, and patients on high-dose statin treatment. |

**Supplementary Table (4). Final Recommendations and Voting Results.**

| Recommendations | Voting Result |
| --- | --- |
| Cardiovascular Risk Assessment |  |
| Consider lipid screening at least once between ages 9 and 11. | 89% |
| Consider lipid screening later again between ages 17 and 21. | 100% |
| Consider lipid screening at age 2-8 years in children with a positive family history of familial dyslipidemia and/or premature ASCVD or other cardiovascular risk factors such as diabetes, hypertension, obesity, or other conditions that accelerate atherosclerotic risk. | 95% |
| Consider risk factor evaluation to assign risk status during the first patient visit for adults aged 20 years and older. Depending on the results, initiate efforts to control risk factors with re-evaluation or initiate lifestyle changes. | 100% |
| In Egypt, use the very high-risk SCORE2 tools to calculate the estimated CVD risk within the next 10 years for apparently healthy people aged 40-69 years (and SCORE2-OP for older individuals) without established ASCVD, DM, CKD, and genetic/rarer lipid disorders. | 95% |
| Patients at very high risk with at least one of the following criteria should be considered at extreme CVD risk: 1) multiple major CV events, especially if recurrent within two years, 2) one major CV event and 2 or more high-risk conditions such as current smoking, DM, HTN, and CKD, 3) polyvascular disease, 4) multivessel CAD, and 5) recent ACS within the past 12 months. | 100% |
| For patients in the moderate risk category, it is advisable to check for risk modifiers. | 100% |
| Laboratory Measurement of lipids and Lipoproteins |  |
| Measure either a fasting or a non-fasting plasma lipid profile, including TC, TG, high-density lipoprotein cholesterol (HDL-C), LDL-C, and non-HDL-C, to estimate ASCVD risk. | 95% |
| Perform a repeat fasting lipid profile if an initial non-fasting lipid profile reveals triglyceride levels of 400 mg/dL or higher for assessment of fasting triglyceride levels and baseline LDL-C. | 100% |
| Perform a 12-hour fasting plasma lipid profile in those with a history of familial dyslipidemia as well as in individuals with known high triglyceride levels. | 100% |
| Lp(a) measurement may be considered at least once in each adult person's lifetime to identify those with very high inherited Lp(a) levels >180 mg/dL, especially in those with a family history of premature CVD or genetic dyslipidemia. | 100% |
| Lp(a) should be considered in patients with premature ASCVD and recurrent vascular events despite LDL-C and non-HDL-C being at target levels and as a risk modifier in individuals at moderate and high risk. | 100% |
| Therapeutic Goals and Regimens |  |
| Lifestyle modification is advised for primary or secondary prevention for all individuals, whether they are eligible for lipid-lowering therapy or not. | 100% |
| In patients who are at low or moderate risk with LDL-C levels above the respective goals (but < 190 mg/dL), a 3-month trial of lifestyle intervention is recommended followed by re-assessment of lipid profile. | 89% |
| In both primary and secondary preventive measures for individuals at very high and extreme CVD risk, a therapeutic regimen that achieves ≥ 50% LDL-C reduction from baseline and an LDL-C goal of < 55 mg/dL are recommended. | 100% |
| In patients at high risk, a treatment regimen that achieves ≥50% LDL-C reduction from baseline and an LDL-C goal of < 70 mg/dL is recommended. | 95% |
| In individuals at moderate risk, an LDL-C goal of < 100 mg/dL should be taken into consideration. | 100% |
| In persons who are at low risk, an LDL-C goal < 116 mg/dL may be considered. | 95% |
| Non-HDL-C of < 85, 100, and 130 mg/dL should be considered as secondary goals for extreme and very high-, high-, and moderate-risk individuals, respectively. | 100% |
| Monitoring of Lipids and Enzymes in Patients on Lipid-Lowering Therapy |  |
| Assess the response to pharmacological lipid-lowering therapy 4-12 weeks after initiation or adjustment of therapy to evaluate treatment effectiveness and any necessary adjustments. In acute coronary syndrome, assessment of the response should be done earlier, after 4-6 weeks. | 100% |
| After a stable LDL-C goal is achieved, regular lipid monitoring is recommended every six months for patients at extreme CV risk and annually for patients in the other risk groups. | 100% |
| During follow-up, a full lipid profile should be performed. Additionally, non-HDL-C should also be assessed and used as a secondary treatment target. | 100% |
| Before starting statin therapy, patients should be evaluated for potential predisposing factors for statin-associated side effects. | 100% |
| Alanine transaminase (ALT) should be routinely measured in patients on statins before treatment, and in those with normal baseline levels, it should be repeated once 4-6 weeks after starting drug treatment or after a dose increase. Otherwise, routine ALT measurement is not recommended unless symptoms suggesting liver disease evolve. Control of ALT is still recommended during treatment with fibrates. | 100% |
| If ALT rise is less than 3 times the upper limit of normal, continue therapy and re-check liver enzymes in 4-6 weeks. | 100% |
| If ALT rise is 3 or more times the upper limit of normal, discontinue lipid-lowering therapy or decrease dose and re-check liver enzymes within 4-6 weeks. Cautious re-initiation of therapy may be considered after ALT has returned to normal with frequent rechecking of ALT levels. | 100% |
| Creatine kinase (CK) should be routinely measured in patients who will receive statins before treatment and after dose increases. If the baseline CK rise is more than four times the upper limit of normal, do not initiate drug therapy and re-check. Otherwise, routine CK measurement is not recommended unless the patient develops myalgia. | 95% |
| If CK becomes elevated in a person taking lipid-lowering drugs, first re-evaluate the indication for statin treatment. Further management would depend on whether the patient has muscle symptoms and the degree of CK elevation. | 100% |
| If CK rises >10 fold the upper limit of normal, discontinue treatment, check renal function, and monitor CK every 2 weeks. | 100% |
| If CK levels rise between 4 and 10 times the upper limit of normal and the patient is symptomatic, discontinue statin and monitor CK. | 100% |
| If CK levels rise between 4 and 10 times the upper limit of normal and the patient is asymptomatic, continuing the statin while re-checking CK within 2 to 6 weeks should be considered. However, the continuation of therapy is a call of judgment depending on multiple factors, including the patient's overall level of risk. According, you may consider stopping or reducing the dose or changing the type of statin with a follow-up of CPK. | 100% |
| If CK rises <4 fold the upper limit of normal, and the patient is not symptomatic, continue statin, explain to the patient to be alert about muscle symptoms, and re-check CK. | 100% |
| If CK rises <4 fold the upper limit of normal, and the patient has muscle symptoms, monitor symptoms and CK regularly. If symptoms persist, discontinue statin and re-evaluate symptoms after 2-6 weeks. | 95% |
| If CK normalizes after statin discontinuation, consider re-challenge with the same or another statin, low-dose statin, alternate day or once/twice weekly dosing regimen, or combination therapy with a lower statin dose or an alternative drug. | 95% |
| If a statin-based regimen is not tolerated at any dosage, even after rechallenge, bempedoic acid/ezetimibe combination should be considered, with the possible addition of PCSK9 inhibitor, if the goal is not achieved. | 100% |
| In patients who are elderly, at risk of myopathy, have CKD, or have partial statin intolerance, consider the initiation with a combination of moderate-intensity statin and ezetimibe. | 95% |
| In the case of complete statin intolerance, an initial combination of ezetimibe and bempedoic acid can be considered. Further addition of PCSK9 targeted therapy may be needed according to the LDL-C level achieved. Consider the possibility of transient CK elevation for other reasons, such as exertion or intramuscular injections. If CK remains elevated, consider myopathy. | 100% |
| HbA1c or blood glucose should be checked at least annually in patients at high risk of developing diabetes, such as the elderly, patients with metabolic syndrome, obesity, or other signs of insulin resistance, and patients on high-dose statin treatment. | 100% |
| Management of Dyslipidemia |  |
| Statins should be prescribed, when indicated, up to the highest tolerated dose to achieve LDL-C goals based on the specific risk level, with exceptions for elderly patients, those with CKD, and those intolerant to statins, who should be managed on an individual basis. | 100% |
| In patients who do not achieve LDL-C goals with the maximum tolerated dose of a statin, the addition of ezetimibe to the statin regimen is recommended. | 100% |
| For primary prevention patients at very-high risk without FH, if the LDL-C goal is not achieved on a maximum tolerated dose of a statin and ezetimibe, a combination with a PCSK9 targeted therapy may be considered. | 100% |
| For secondary prevention patients at very-high risk who do not achieve their goal on a maximum tolerated dose of a statin and ezetimibe, a combination with a PCSK9 targeted therapy is recommended. | 100% |
| For very-high-risk FH patients (i.e. with ASCVD or with another major risk factor) who do not achieve their goal on a maximum tolerated dose of a statin and ezetimibe, a combination with a PCSK9 targeted therapy is recommended. | 100% |
| In patients who do not tolerate statin-based regimens at any dosage, even after rechallenge, the use of bempedoic acid in combination with ezetimibe should be considered, with the potential addition of PCSK9 inhibitor therapy if LDL-C targets are not met. | 100% |
| In patients who are elderly, at risk of myopathy, have CKD, or have partial statin intolerance, consider the initiation with a combination of moderate-intensity statin and ezetimibe. | 95% |
| An initial combination of ezetimibe and bempedoic acid can be considered. Depending on the LDL-C level achieved, further addition of PCSK9 targeted therapy may be needed in case of a patient with statin intolerance. | 95% |
| Identify individuals at extremely high ASCVD risk from the rest of the very high-risk category. | 100% |
| In individuals with extremely high ASCVD risk, consider a combination therapy with a high-intensity statin, if tolerated, and ezetimibe as an initial approach. | 100% |
| The LDL-C target in individuals with extremely high ASCVD risk should be below 55 mg/dL, with consideration of a lower initial target of below 40 mg/dL on an individual basis. | 100% |
| In individuals with extremely high ASCVD risk, if additional LDL-C lowering of less than 20% is required, bempedoic acid can be considered. If a greater reduction is required, PCSK9-targeted therapy should be considered. | 100% |
